# Cardiac Remodeling After Myocardial Infarction: Functional Contribution of microRNAs to Inflammation and Fibrosis

**DOI:** 10.3389/fcvm.2022.863238

**Published:** 2022-04-13

**Authors:** Fahimeh Varzideh, Urna Kansakar, Kwame Donkor, Scott Wilson, Stanislovas S. Jankauskas, Pasquale Mone, Xujun Wang, Angela Lombardi, Gaetano Santulli

**Affiliations:** ^1^Department of Medicine, Einstein-Mount Sinai Diabetes Research Center (ES-DRC), Albert Einstein College of Medicine, Fleischer Institute for Diabetes and Metabolism (FIDAM), Einstein Institute for Aging Research, New York, NY, United States; ^2^Department of Molecular Pharmacology, Albert Einstein College of Medicine, Wilf Family Cardiovascular Research Institute, Institute for Neuroimmunology and Inflammation (INI), New York, NY, United States

**Keywords:** cardiac remodeling, clinical trials, drug development, epigenetics, heart failure, ischemic heart disease, non-coding RNA, oxidative stress

## Abstract

After an ischemic injury, the heart undergoes a complex process of structural and functional remodeling that involves several steps, including inflammatory and fibrotic responses. In this review, we are focusing on the contribution of microRNAs in the regulation of inflammation and fibrosis after myocardial infarction. We summarize the most updated studies exploring the interactions between microRNAs and key regulators of inflammation and fibroblast activation and we discuss the recent discoveries, including clinical applications, in these rapidly advancing fields.

## Introduction

microRNAs (also known as miRNAs or miRs) are small (∼22 nucleotides) non-coding RNA molecules that can regulate gene expression *via* translational repression and/or post-transcriptional degradation; they have been implied in a number of cardiovascular disorders (1–4). Following myocardial infarction (MI), the heart undergoes a series of structural, functional, and pathophysiological modifications that are commonly known as cardiac remodeling (5–7).

In this minireview, we will focus on the role of miRNAs in two specific components of post-ischemic cardiac remodeling, namely fibrosis and inflammation (Table 1).

**TABLE 1:** miRNAs and their target genes involved in inflammation and fibrosis post-MI.

miRNA	Target gene(s)	References
**Inflammation**		
miR-19a/b	Bim1/PTEN	(8)
miR-21	KBTBD7/NF-κB	(9)
miR-22	VE-cadherin	(10)
miR-92a	JNK/ERK1/2	(11)
miR-133a	VEGFR2 and FGFR1	(12)
miR-144-3p	PI3K/Akt/VEGF	(13)
miR-144-3p	PTEN	(13, 14)
miR-146a-5p	TLR7	(15)
miR-155	SOCS1	(16)
miR-320	PI3K/Akt/VEGF	(13)
miR-375	PDK-1/Akt	(17)
**Fibrosis**		
miR-1	Cyclin D2 and CDK6	(18)
miR-1	PTEN/Akt	(19)
miR-19b	PTEN	(20)
miR-21	TGF-β1/SMAD7	(21)
miR-21	CADM1/STAT3	(22)
miR-21	Notch/Jagged1	(23)
miR-22	Osteoglycin/VSMC marker genes	(24)
miR-34a	SMAD4	(25)
miR-92a	SMAD7	(26)
miR-125b	p53/TGF-β1	(27)
miR-126	HIF-1α	(28, 29)
miR-130a	PTEN/Akt	(30)
miR-132	FOXO3 and SERCA2a	(49)
miR-133a	GTP Cyclohydrolase 1 (GCH1)	(31, 32)
miR-144-3p	PTEN	(14)
miR-146b-5p	IRAK1 and CEACAM1	(33)
miR-155	Ang II	(34)
miR-195	SMAD7	(35)
miR-200a-3p	PIGF/VEGF-A	(36)
miR-214	Mfn2	(37)
miR-590-3p	ZEB1	(38)

## Effects of microRNAs on Cardiac Fibroblasts Post-Mi

Cardiac fibroblasts are the most abundant interstitial cell type in the heart (39–43). They play essential roles in the regulation of cardiac remodeling following an ischemic injury; indeed, they are generally activated in response to pathological stress or injury, and start to proliferate quickly and to produce extracellular matrix (ECM), eventually leading to cardiac fibrosis (18, 42, 44–46). Activated cardiac fibroblasts, known as myofibroblasts, exhibit an increased proliferation rate and migratory capacities (47, 48).

The miRNA-212/132 family was originally detected by Ucar et al. (49); miR-132 has been later shown to fine-tune Angiotensin II actions in cardiac fibroblasts (50). These observations led to a clinical trial (51), which will be discussed in detail in the last paragraph of this minireview.

Another miRNA, generally considered to be muscle-specific (52, 53), namely miR-1, was shown to be expressed in cardiac fibroblasts as well, and to be significantly down-regulated upon their activation (18); miR-1 negatively regulates cardiac fibroblast proliferation by targeting Cyclin D2 and CDK6 (18). Glass and Singla demonstrated that miR-1 triggers cardiac differentiation and ameliorates heart function *via* targeting the PTEN/Akt pathway (19). Likewise, miR-19b (20) and miR-144-3p (14) have been proven to regulate proliferation and migration of cardiac fibroblasts by modulating PTEN expression.

By specifically targeting the signaling pathway that includes transforming growth factor β1 (TGF-β1) and mothers against DPP homologs 7 (SMAD7), miR-21 has been validated as an activator of cardiac fibroblasts post-MI, subsequently eliciting cardiac fibrosis, as well (23, 54); corroborating these findings, miR-21 had been previously shown to upregulate the expression of α-smooth muscle actin (α-SMA), Col-1, and F-actin (21) and to promote fibroblast proliferation and interstitial fibrosis *via* targeting the CADM1/STAT3 signaling pathway (22); on the other hand, miR-21 suppression reduces cardiac fibroblast proliferation (22). Independent investigators have confirmed that miR-21 expression is upregulated by TGF-β1 and mediates the conversion of quiescent cardiac fibroblasts to activated myofibroblasts *via* targeting the Notch/Jagged1 pathway (23, 55, 56), and that miR-21 is strategic in mediating the profibrotic role of cardiac macrophages (57).

Our group was the first to demonstrate that two different miRs, namely miR-92a (26) and miR-195 (35), act as transcriptional regulators of SMAD7, an inhibitor of α-SMA, which is a well-established marker of myofibroblast activation (58). We found that miR-92a is significantly upregulated in cardiomyocyte-derived exosomes and in fibroblasts isolated after MI compared with SHAM conditions, indicating that miR-92a is transferred to fibroblasts in form of exosomal cargo and is essential for the activation of cardiac myofibroblast (26). We also observed (35) that miR-195, a cardiomyocyte-specific miRNA that is upregulated in cardiac myocytes after an ischemic insult (59), is secreted by injured cardiomyocytes within cardiac exosomes (cardiosomes) and transferred to fibroblasts, where it relieves the SMAD7-mediated inhibition of α-SMA transcription, eventually leading to myofibroblast phenoconversion (35). The mechanistic involvement of exosomal miRs in cardiac fibroblasts has been more recently also reported by Suresh Verma’s research team, who determined that TGF-β1 activates cardiac fibroblasts and myofibroblasts-derived exosomes causes endothelial dysfunction mediated by miR-200a-3p *via* PIGF/VEGF-A signaling pathway (36).

Yuan and coworkers demonstrated that miR-590-3p can decrease proliferation, differentiation, and migration of cardiac fibroblasts *via* targeting ZEB1 expression (38); substantiating these observations, inhibiting miR-590-3p drastically augmented proliferation and migration of cardiac fibroblasts (38). Jazbutyte and colleagues revealed that miR-22 upregulation accelerates the senescence of cardiac fibroblasts by targeting osteoglycin (also known as mimecan) (60). Other reports have also indicated that miR-22 upregulates some specific genes of vascular smooth muscle cells (VSMC), thereby suppressing VSMC proliferation and migration, as well (24, 61–63).

Notably, miR-34a modulates cardiac fibrosis after MI *via* targeting SMAD4 (25): the upregulation of miR-34a promotes the profibrogenic activity of TGF-β1 in cardiac fibroblasts, whereas suppressing miR-34a has opposite effects (25). Similarly, miR-125b is decisive for the induction of cardiac fibrosis and plays a critical role in inducing fibroblast proliferation by suppressing p53 (27), a growth regulator and anti-fibrotic factor (64, 65). TGF-β1 changes the morphology of fibroblasts from spindle-shaped to well-spread myofibroblast-like cells and causes upregulation of molecular markers of myofibroblast activation, such as α-SMA and Col1; miR-125b was found to be overexpressed in endothelial-to-mesenchymal transition (EndMT)-derived myofibroblast-like cells, and such upregulation, triggered by TGF-β1, causes the inhibition of anti-fibrotic genes thus promoting the proliferation and activation of cardiac fibroblasts, leading to fibrosis (27). The inhibition of miR-155 has been reported to decrease the conversion of fibroblasts to myofibroblasts and to improve the cardiac fibrotic remodeling induced by Angiotensin II (34). Another miRNA that was shown to regulate fibroblast survival and proliferation *via* targeting the mitofusin-2 (Mfn2) gene is miR-214 (37).

A very recent study by Liao et al. has shown that the upregulation of miR-146b-5p activates fibroblast proliferation, migration, conversion of fibroblast to myofibroblast, and endothelial cell dysfunction (33); in contrast, inhibition of miR-146b-5p has opposite effects and promotes angiogenesis by targeting IRAK1 and CEACAM1 (33). Thus, suppression of miR-146b-5p may be a novel therapeutic approach to treat cardiac fibrotic dysfunction after MI.

## Cardiac Inflammation and microRNAs

One of the most studied miRNAs in the regulation of the post-ischemic inflammatory response in the heart is miR-21, which is known to attenuate excessive inflammation and cardiac dysfunction after MI by targeting MKK3/6 and suppressing p38 and NF-κB signaling activation post-MI (9) and to stimulate MAP kinase signaling in fibroblasts (66), whereas its deficiency induces inflammatory reactions post-MI and significantly augments the phosphorylation of p38, IKKα/β, and p65 (9). Of note, miR-21 is also upregulated in cardiac macrophages (57), and nanoparticle-based targeted delivery of miR-21 to cardiac macrophages has been shown to ameliorate cardiac remodeling post-MI, modifying the phenotype of macrophages from a pro-inflammatory to a reparative state (67).

Right after MI, the expression levels of many pro-inflammatory cytokines including IL-1β, IL-6, and TNF-α increase, contributing to cardiac remodeling (68); miR-146a-5p induces expression of pro-inflammatory cytokines including CXCL2, IL-6, and TNF-α, and activates innate immune cells such as CD45^+^ leukocytes, Ly6C^mid+^ monocytes, Ly6G^+^ neutrophils *via* a TLR7-dependent mechanism (15). Moreover, miR-146a-5p causes cardiac endothelial barrier dysfunction, further triggering an increased transmigration of monocytes and neutrophils into the myocardium (15). The inhibition of miR-146b-5p considerably increases cytokines such as IL-1β, IL-6, TNF-α, and MCP-1. In addition, *in vivo* assays demonstrated that CD206^+^ macrophages are increased due to suppression of miR-146b-5p (33).

Reducing the overexpression of miR-155 modulates the expression of cytokines such as IL-1 and CXCL8 (69); miR-155-enriched exosomes slow down cardiac fibroblast proliferation by downregulating Son of Sevenless 1 (SOS1) expression—which is also involved in the regulation of inflammation (70)—and can promote inflammation and atherosclerotic lesions by increasing STAT3 and NF-κB *via* targeting Suppressor of Cytokine Signaling 1 (SOCS1) expression (16). By conducting *in vivo* experiments. Wang et al. observed increased fibroblast proliferation, augmented collagen production, and reduced cardiac inflammation in the hearts of miR-155-deficient mice compared to control animals (71). The expression of miR-155 is also upregulated in exosomes of activated cardiac macrophages post-MI (72, 73).

Another miR fundamental in post-MI remodeling is miR-22: its overexpression triggers the synthesis of proinflammatory cytokines such as IL-1β, IL-6, and IL-8 (74), moreover, the same miR-22 is able to regulate inflammation and angiogenesis by specifically targeting VE-cadherin (10).

The synergistic interplay between inflammation and angiogenesis is crucial in post-ischemic cardiac remodeling and healing (75–80), and several researchers have demonstrated that non-coding RNAs are involved in the regulation of both these processes (78, 81). For instance, miR-133a has been shown to have beneficial effects on infarcted hearts by inhibiting inflammation and angiogenesis *via* FGFR1 and VEGFR2 signaling pathways (82, 83). Similarly, miR-320 and miR-144-3p, have been shown to be involved in post-MI responses by regulating PTEN/PI3K/Akt signaling pathway (13, 84, 85); miR-144-3p promotes cardiac fibrosis *via* targeting PTEN following MI (14); miR-199a-3p and miR-590-3p also improve cardiac function after MI (8, 86); miR-19a/19b inhibits the inflammatory response and has been shown to enhance cardiac function post-MI by targeting Bim1 and PTEN (8). All these results are relevant when considering that *in vivo* studies carried out in infarcted mice revealed that angiogenesis can be improved by inhibiting PTEN via activating the PI3K/Akt/VEGF pathway (13, 87, 88). On the same line, Lu and coworkers reported that the overexpression of miR-130a promotes endothelial cell proliferation and migration by increasing Akt phosphorylation and inhibiting PTEN (30); the same group also demonstrated that the activation of PI3K/Akt signaling enhances angiogenesis and decreases the progression of MI and fibrosis, attenuating myocardial dysfunction and reducing the risk of cardiac rupture post-MI (30). Several members of the miRNA cluster 17∼92 regulate angiogenesis following MI (89). Equally important, suppressing miR-375 was shown to mitigate post-MI inflammatory responses while improving angiogenesis *via* PDK-1/Akt signaling mechanisms (17).

Endothelial cells play decisive roles in post-MI cardiac remodeling (90, 91), and miR-126 is considered one of the most important miRs in endothelial biology (92–94). In mature endothelial cells, miR-126 promotes vascular homeostasis by preventing angiogenesis and preserving the quiescent endothelial phenotype via the HIF-1α pathway (28, 29). Remarkably, miR-199a-5p inhibition causes upregulation of VEGF-A, enhances nitric oxide (NO) bioavailability by activating eNOS (endothelial NO synthase), and stimulates the formation of network-like structures (95). Likewise, miR-133a causes endothelial dysfunction by suppressing eNOS, and its overexpression significantly reduces endothelial cell survival by targeting GTP Cyclohydrolase 1 GCH1 (31, 32). Lastly, the overexpression of miR-92a inhibits endothelial cell migration and regulates angiogenesis (11, 89) whereas its inhibition enhances endothelial cell proliferation *via* the activation of the JNK and ERK1/2 pathway (11).

## Clinical Perspective: microRNA-Based Drug Development

miRNA-based therapeutics have been proven to be effective for treating cardiovascular diseases (1, 96). Since miRNAs can regulate multiple genes using different signaling pathways, they have a great potential as novel therapeutic agents; therapeutic strategies based on miRNA modulation have been widely utilized in angiogenesis, atherosclerosis, ischemic injury, vascular remodeling, hypertrophy, and fibrosis (97, 98).

**FIGURE 1:**
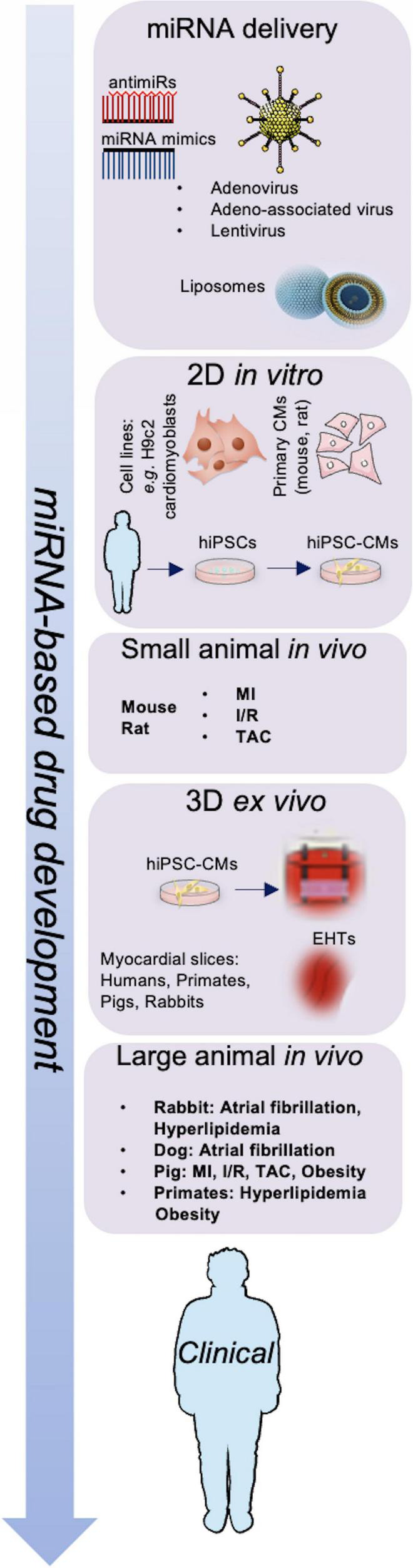
Schematic representation of miRNA-based drug development. Adenovirus, adeno-associated virus (AAV), lentivirus particles, and liposomes are used to deliver miRNA mimics or antimiRs; miRNAs are investigated *in vitro*, *in vivo*, and *ex vivo* models to develop next-generation therapeutics for cardiovascular diseases. CMs, cardiomyocytes; EHTs, engineered heart tissues; hiPSCs, human induced pluripotent stem cells; I/R, ischemia-reperfusion; MI, myocardial infarction; TAC, transverse aortic constriction.

Treatment options with miRNA-based drugs include suppression of miRNAs to reduce the levels of upregulated miRNAs and substitute missing miRNA to restore the expression of miRNAs in post-ischemic HF (99). A representation of the work-flow leading to miRNA-based drug development is shown in Figure 1. Several approaches to deliver miRNAs to specific target tissues or organs without degradation have been discovered including viral vectors, vesicles, antagomirs or mimics, plasmids and sponges, with a focus on bioavailability and bio-efficacy (100).

In recent years, miRNA-targeted therapeutics have been tested in clinical trials, mostly in cancer; because of the limited space allowed in this minireview, for these aspects we refer to dedicated reviews (101–104). A successful example of how to develop a miRNA-based therapy in cardiovascular medicine is given by miR-132, which has among its targets FOXO3 and SERCA2a (49, 105).

Several *in vitro* and *in vivo* experiments demonstrated that inhibiting miR-132 caused a reduction of cardiac fibrosis, normalization of autophagy, and calcium signaling, and reversal of cardiomyocyte hypertrophy; after a pharmacokinetic assessment, miR-132 inhibition was shown to improve HF in a clinically relevant pig model (96, 106). The following logical step was the clinical investigation: a prospective, randomized, and placebo-controlled phase 1b dose-escalation study was designed to assess safety, pharmacokinetics, target engagement, and exploratory pharmacodynamic effects of miR-132 inhibition, achieved by administering a chemically modified oligonucleotide (CDR132L) containing locked nucleic acid (LNA) nucleotides and phosphonothioate linkages to increase *in vivo* stability (51). The trial, conducted in patients with stable chronic HF of ischemic origin (20 randomized to CDR132L and 8 to placebo), revealed that CDR132L was overall safe and well-tolerated, confirmed linear plasma pharmacokinetics with no signs of accumulation, and, despite the small size, suggested cardiac functional improvements, reflected in a clinically meaningful median reduction in NT-proBNP and narrowing of the QRS complex (51).

## Conclusion

In this review, we have presented the most updated investigations on microRNAs and some primary regulators of inflammation and fibrosis, also discussing the most recent discoveries and actual applications in the clinical scenario.

## Author Contributions

GS: conceptualization and supervision. FV and UK: writing—original draft preparation. KD, SW, SSJ, PM, XW, AL, and GS: writing—review and editing. All authors listed have made a substantial, direct, and intellectual contribution to the work, and approved it for publication.

## Conflict of Interest

The authors declare that the research was conducted in the absence of any commercial or financial relationships that could be construed as a potential conflict of interest.

## Publisher’s Note

All claims expressed in this article are solely those of the authors and do not necessarily represent those of their affiliated organizations, or those of the publisher, the editors and the reviewers. Any product that may be evaluated in this article, or claim that may be made by its manufacturer, is not guaranteed or endorsed by the publisher.
